# Health impact and cost-effectiveness of expanding routine immunization coverage in India through Intensified Mission Indradhanush

**DOI:** 10.1093/heapol/czae024

**Published:** 2024-04-03

**Authors:** Emma Clarke-Deelder, Christian Suharlim, Susmita Chatterjee, Allison Portnoy, Logan Brenzel, Arindam Ray, Jessica L Cohen, Nicolas A Menzies, Stephen C Resch

**Affiliations:** Department of Global Health and Population, Harvard T. H. Chan School of Public Health, Boston, MA 02115, United States; Department of Epidemiology and Public Health, Swiss Tropical & Public Health Institute, Allschwil 4123, Switzerland; University of Basel, Basel 4001, Switzerland; Center for Health Decision Science, Harvard T. H. Chan School of Public Health, Boston, MA 02115, United States; Management Sciences for Health, Medford, MA 02155, United States; Research Department, George Institute for Global Health, New Delhi, Delhi 110025, India; Department of Medicine, University of New South Wales, New South Wales 2052, Australia; Center for Health Decision Science, Harvard T. H. Chan School of Public Health, Boston, MA 02115, United States; Department of Global Health, Boston University School of Public Health, Boston, MA 02118, United States; Bill & Melinda Gates Foundation, Seattle, WA 98109, United States; Bill & Melinda Gates Foundation, New Delhi, Delhi 110067, India; Department of Global Health and Population, Harvard T. H. Chan School of Public Health, Boston, MA 02115, United States; Department of Global Health and Population, Harvard T. H. Chan School of Public Health, Boston, MA 02115, United States; Center for Health Decision Science, Harvard T. H. Chan School of Public Health, Boston, MA 02115, United States; Center for Health Decision Science, Harvard T. H. Chan School of Public Health, Boston, MA 02115, United States

**Keywords:** Child health, health economics, vaccines

## Abstract

Many children do not receive a full schedule of childhood vaccines, yet there is limited evidence on the cost-effectiveness of strategies for improving vaccination coverage. Evidence is even scarcer on the cost-effectiveness of strategies for reaching ‘zero-dose children’, who have not received any routine vaccines. We evaluated the cost-effectiveness of periodic intensification of routine immunization (PIRI), a widely applied strategy for increasing vaccination coverage. We focused on Intensified Mission Indradhanush (IMI), a large-scale PIRI intervention implemented in India in 2017–2018. In 40 sampled districts, we measured the incremental economic cost of IMI using primary data, and used controlled interrupted time-series regression to estimate the incremental vaccination doses delivered. We estimated deaths and disability-adjusted life years (DALYs) averted using the Lives Saved Tool and reported cost-effectiveness from immunization programme and societal perspectives. We found that, in sampled districts, IMI had an estimated incremental cost of 2021US$13.7 (95% uncertainty interval: 10.6 to 17.4) million from an immunization programme perspective and increased vaccine delivery by an estimated 2.2 (−0.5 to 4.8) million doses over a 12-month period, averting an estimated 1413 (−350 to 3129) deaths. The incremental cost from a programme perspective was $6.21 per dose ($2.80 to dominated), $82.99 per zero-dose child reached ($39.85 to dominated), $327.63 ($147.65 to dominated) per DALY averted, $360.72 ($162.56 to dominated) per life-year saved and $9701.35 ($4372.01 to dominated) per under-5 death averted. At a cost-effectiveness threshold of 1× per-capita GDP per DALY averted, IMI was estimated to be cost-effective with 90% probability. This evidence suggests IMI was both impactful and cost-effective for improving vaccination coverage, though there is a high degree of uncertainty in the results. As vaccination programmes expand coverage, unit costs may increase due to the higher costs of reaching currently unvaccinated children.

Key messages

- Few studies have measured the cost-effectiveness of efforts to increase vaccination coverage. Even fewer have measured the cost-effectiveness of approaches for reaching zero-dose children, which is a global priority.
- We estimated the health impact and cost-effectiveness of Intensified Mission Indradhanush (IMI), a large-scale intervention that aimed to improve routine immunization coverage in India in 2018–2019. IMI was an example of ‘periodic intensification of routine immunization’ (PIRI).
- We found that IMI was cost-effective when using a threshold of 1× per capita GDP per disability-adjusted life year (DALY) averted.
- The estimated cost per incremental dose delivered was $6.21 and the estimated cost per zero-dose child reached was $82.99, suggesting that PIRI may be a cost-effective way to improve vaccination coverage and reach zero-dose children in low- and middle-income countries.

## Introduction

Vaccination is often cited as among the most cost-effective public health interventions, but many children do not receive a full schedule of childhood vaccines (Ozawa *et al*., 2017; World Health Organization: Immunization, Vaccines, and Biologicals, 2018). In 2022, 11% of children aged 12–23 months worldwide had not received a single dose of diphtheria-tetanus-pertussis (DTP)-containing vaccine and were therefore considered ‘zero-dose children’ (Kaur *et al*., 2023), with no vaccine-conferred immunity against these diseases. After a decade of very little progress in expanding global coverage of traditional vaccines (World Health Organization: Immunization, Vaccines, and Biologicals, 2018), the COVID-19 pandemic caused significant disruptions to immunization programmes worldwide, leading to sharp reductions in coverage and leaving many children exposed to vaccine-preventable diseases (Causey *et al*., 2021).

To achieve the goal set out in the Global Immunization Agenda 2030 to ‘leave no child behind’, it is critical to identify cost-effective ways to improve coverage and reach zero-dose children (World Health Organization, 2020). However, there is limited evidence on the cost-effectiveness of strategies for increasing coverage (Oyo-Ita *et al*., 2016; Munk *et al*., 2019). A wide range of alternative approaches may be used, including supply-side strategies, such as training health workers or increasing vaccine delivery sites, or demand-side strategies, such as awareness-raising interventions or providing incentives to vaccinate children. In a recent systematic review of the cost-effectiveness of different approaches to improving immunization coverage, estimates ranged from 1.00 USD per incremental child vaccinated against hepatitis B at birth through the use of auto-filled syringes in Indonesia (Levin *et al*., 2005), to 161.95 USD per incremental child vaccinated with DTP3 through an immunization programme strategy focused on raising mothers’ awareness on the benefits of DTP vaccination in Uttar Pradesh, India (Powell-Jackson *et al*., 2018). Evidence on the cost-effectiveness of strategies for reaching zero-dose children is also lacking, even though optimal strategies for reaching zero-dose children (equivalent to increasing the coverage of DTP1) may differ from optimal strategies for increasing coverage of antigens for older children. One study on a health information dissemination intervention in Uttar Pradesh found that the incremental cost per child receiving at least one vaccine dose was 6.68 USD (Pandey *et al*., 2007; Munk *et al*., 2019).

India’s immunization programme is the largest in the world, serving an annual birth cohort of approximately 26 million children. In India in 2016, coverage of traditional vaccines—including one dose of the Bacillus–Calmette–Guérin (BCG) vaccine, three doses of DTP-containing vaccine, three doses of polio vaccine and one dose of measles vaccine—was only 62% (Government of India, Ministry of Family Health and Welfare, International Institute for Population Sciences, 2015–2016).

In this study, we evaluate the cost-effectiveness of a periodic intensification of routine immunization (PIRI) intervention in India. PIRI, a widely applied strategy for increasing routine vaccination coverage, adapts techniques from supplementary immunization activities (SIAs) and applies them to the delivery of routine vaccines (John Snow International, 2009). PIRI interventions are typically time-limited and intermittent, with examples including Child Health Days and National Vaccination Weeks. In contrast with SIAs, which vaccinate all members of the target population regardless of vaccination status, PIRI interventions take prior vaccination history into account, and vaccine doses administered during PIRI interventions are recorded on vaccine cards as routine doses (World Health Organization, 2023).

We focus on the case of IMI, one of the largest-ever PIRI interventions, implemented in India in 2017–2018 (Ministry of Health & Family Welfare, Government of India, 2017). Understanding the cost-effectiveness of IMI can help inform decisions about future implementation of PIRI interventions, which often involve a large mobilization of health system resources. It can also shed light on how the unit costs faced by immunization programme costs may change as programmes expand coverage to hard-to-reach children.

## Materials and methods

This study builds on two previously published studies that estimated the costs of IMI (Chatterjee *et al*., 2021) and the impacts of IMI on vaccine delivery (Clarke-Deelder *et al*., 2021).

Chatterjee *et al*. (2021) estimated the costs of IMI in a sample of 40 participating districts (Chatterjee *et al*., 2021). The sample was drawn from the five Indian states with the highest rates of IMI participation: Assam, Bihar, Maharashtra, Rajasthan and Uttar Pradesh. Within these states, districts were randomly sampled proportionate to their size. Data on the costs of IMI were collected retrospectively from administrative records, financial records and structured interviews at the health facility, sub-district and district levels. Costs included all costs of planning and implementing IMI. Chatterjee et al. found that the cost per dose of IMI ranged from $3.45 in Uttar Pradesh to $12.23 in Maharashtra. These estimates tell us about the cost per dose delivered during an IMI session; this is different from the cost per incremental dose delivered (the focus of the present study) through IMI because it does not account for the fact that some doses delivered through IMI displaced routine doses. We therefore build on Chaterjee et al. by combining cost estimates with impact estimates from Clarke-Deelder et al. (2021) that account for this displacement.

Clarke-Deelder *et al*. (2021) estimated the incremental effects of IMI on vaccine delivery across all participating districts using a quasi-experimental analysis (Clarke-Deelder *et al*., 2021). Controlled Interrupted Time-Series (CITS) regression models were fit to data on vaccine delivery volumes from India’s Health Management & Information System (HMIS). Clarke-Deelder et al. found that IMI led to a significant increase in vaccine delivery volumes during the 4-month implementation period: across 13 infant vaccines included in the study, the median effect size was an improvement of 10.6%. After implementation ended, monthly vaccine delivery volumes appeared to return to their pre-IMI levels, suggesting that the benefits of IMI were one-off and the programme objective of having a sustained impact was not achieved.

In this study, we build on this previous work by using a mathematical model (Walker *et al*., 2013) to translate estimates of incremental doses delivered to health impact estimates, and applying a cost-effectiveness framework. All estimates in this study are for the sample of 40 districts analysed by Chatterjee *et al*. (2021).

### Study setting and intervention

India’s vaccination programme delivers vaccines to children and pregnant women for free in public health facilities and through outreach services. The programme has made significant progress in increasing coverage in recent decades: from 1992 to 2016, coverage of DTP3 in India increased from 47% to 78%, and coverage of DTP1 increased from 62% to 90% [Bombay: International Institute for Population Sciences (IIPS) and ICF, 1992–1993; Government of India, Ministry of Family Health and Welfare, International Institute for Population Sciences, 2015–2016]. However, India remains the home to the second largest number of zero-dose children worldwide (after Nigeria), and children from disadvantaged backgrounds, including children in lower-income households, rural areas and with less educated mothers, are more likely to fall in this group (Johri *et al*., 2021).

IMI was implemented from October 2017 through January 2018 with the goal of increasing coverage of routine vaccines in selected districts with low immunization coverage or large numbers of under-immunized children (Gurnani *et al*., 2018). IMI began with door-to-door surveys to identify under-immunized children and inform the selection of IMI implementation sites. Social mobilization was then conducted to raise awareness of the intervention. Finally, during the 4-month implementation period, immunization sessions were conducted for 7 consecutive days per month at the selected sites. IMI focused on the delivery of all routine vaccines for children under the age of 2 and for pregnant women in the routine immunization schedule. This included: the Hepatitis B birth dose; the Bacillus–Calmette–Guérin vaccine; four doses of diphtheria, tetanus and pertussis-containing vaccine; five doses of oral polio vaccine (OPV); two doses of inactivated polio vaccine; two doses of measles-containing vaccine; three doses of rotavirus vaccine; three doses of pneumococcal vaccine; two doses of Japanese Encephalitis vaccine (in endemic areas) and the first and second dose (or booster) of tetanus toxoid vaccine for pregnant women (Supplementary Table S2). Approximately 6 million children and 1 million pregnant women were vaccinated during IMI sessions (Gurnani *et al*., 2018). In this study, we compare IMI with the status quo (no IMI).

### Sample

This study was conducted in a sample of 40 districts in Assam, Bihar, Maharashtra, Rajasthan and Uttar Pradesh. These states were selected because they represented the locations with the greatest number of districts implementing IMI. They are also home to a large concentration of zero-dose children in India (Johri *et al*., 2021). Within the sampled states, districts participating in IMI were randomly sampled for the study using a multi-stage sampling approach (Chatterjee *et al*., 2021).

### Measurement of IMI costs

Data on the incremental economic costs of IMI were collected at the district level, sub-district level and health facility level. Cost data were collected from the immunization programme perspective using structured questionnaires during interviews with programme officials and auxiliary nurse-midwives (ANMs). Sub-centre-level cost data were imputed for two districts where these data could not be collected due to nursing strikes at the time of data collection. Data collection included the costs of vaccines as well as all activities related to the planning and implementation of IMI (e.g. head count surveys to identify unvaccinated children, vaccine transport and alternate vaccine delivery, communication, training, meetings, incentives for health care providers, printing, waste management, supervision, microplanning, mobile teams and mobility support). Costs incurred by recipients (such as the cost of reaching an immunization site) were excluded. The costs of vaccines and injection supplies were calculated based on UNICEF cost estimates (UNICEF, 2020) and included in the main results. Cost data were collected for the period of intervention planning and implementation (2017 through early 2018) to capture all costs associated with the intervention. All costs are presented in 2021 US dollars (USD). Further details on cost data were published previously (Chatterjee *et al*., 2021).

### Measurement of operational and health outcomes

To facilitate comparisons with not only vaccine-related interventions but also other health interventions, our analysis focused on six outcomes: (1) vaccination doses delivered; (2) zero-dose children reached; (3) deaths averted of children under the age of 5; (4) years of life saved; (5) disability-adjusted life-years (DALYs) averted; and (6) costs-of-illness averted. We discounted future costs and health outcomes at 3% in line with the International Decision Support Initiative reference case, and also presented results without discounting as a secondary analysis (Wilkinson *et al*., 2016).

#### Measurement of the impact of IMI on vaccine doses delivered

Since IMI was not implemented in a randomized manner, we used CITS regression—a quasi-experimental design—to estimate the impact of IMI on vaccine delivery (Clarke-Deelder *et al*., 2021). Using data from India’s Health Management Information System (HMIS), we modelled time trends in vaccine delivery for districts that participated in IMI compared to districts that did not participate. The key assumption of this analysis is that, if IMI had not occurred, the trends in participating and non-participating districts would have changed over time in the same way. This approach is preferred to using primary data on the number of vaccine doses delivered during IMI sessions because it accounts for the possibility that IMI displaced routine vaccination (i.e. if children vaccinated during IMI sessions would have otherwise been vaccinated during regular immunization sessions even if IMI had not been implemented).

We fit separate regression models for each childhood vaccine and generated predictions from the fitted models to estimate the number of incremental doses of each vaccine delivered in the 40 districts in the study sample. We estimated the impact of IMI on vaccine delivery over a 1-year period starting with the 4-month IMI implementation period. Our analysis included all vaccines administered to children up to 2 years of age (Supplementary Table S1), excluding those for which data were not available in the HMIS for the full study period (rotavirus, pneumococcal, inactivated polio virus and Japanese encephalitis). For these vaccines, we assumed that the impact of IMI was the same as the impact of IMI on the dose of the pentavalent vaccine (i.e. DTP-hepatitis B-*Haemophilus influenzae* type B) delivered at the same point in the vaccination schedule.

#### Measurement of the impact of IMI on zero-dose children

To estimate the impact of IMI on zero-dose children, we calculated the incremental number of doses of DTP1 delivered in the 40 districts in our sample, using the same methods as for the overall dose calculations.

#### Measurement of the impact of IMI on under-5 deaths averted

We used the Lives Saved Tool (LiST) to estimate the impact of IMI on child mortality. LiST is a publicly available mathematical model that estimates the impact of health interventions on child health outcomes (Walker *et al*., 2013). We generated projections of the number of under-5 deaths during 2018–2027 with and without IMI to calculate the incremental impact. We used all default parameters within LiST for demographic projections and vaccine effectiveness. We estimated coverage improvements attributable to IMI by dividing incremental vaccine doses delivered by estimates of the target population size—assuming population projections from the Census of India, as well as World Bank estimates of the birth rate and the neonatal mortality rate (Census of India, 2020; World Bank, 2020)—and then changed vaccine coverage parameters in the model to reflect this impact. Key parameters included in the analysis are shown in Table 1. This analysis included all vaccines administered to children up to 2 years of age, except for the DTP booster and the OPV booster, which are not included in LiST.

**Table 1.** Study parameters

Study parameter	Value	Source
Discount rate	3%	IDSI Reference Case
Gross Domestic Product per Capita (India, 2020)^a^	$1927	World Bank Databank
*Population parameters*		
Population (Assam, 2017)	33 543 000	Census of India
Population (Bihar, 2017)	115 957 000	Census of India
Population (Maharashtra, 2017)	119 869 000	Census of India
Population (Rajasthan, 2017)	75 248 000	Census of India
Population (Uttar Pradesh, 2017)	219 051 000	Census of India
Crude birth rate (India, 2017)	18.083	World Bank Databank
Neonatal mortality rate (India, 2017)	23.7	World Bank Databank
Gross Domestic Product per capita (India, 2017)	$1997	World Bank Databank
Life expectancy at 1 year old (Assam)	67.7	Census of India
Life expectancy at 1 year old (Bihar)	70.3	Census of India
Life expectancy at 1 year old (Maharashtra)	72.7	Census of India
Life expectancy at 1 year old (Rajasthan)	71.1	Census of India
Life expectancy at 1 year old (Uttar Pradesh)	68.1	Census of India
Life expectancy at 5 years old (Assam)	64.8	Census of India
Life expectancy at 5 years old (Bihar)	67.1	Census of India
Life expectancy at 5 years old (Maharashtra)	68.8	Census of India
Life expectancy at 5 years old (Rajasthan)	67.6	Census of India
Life expectancy at 5 years old (Uttar Pradesh)	64.8	Census of India
*Vaccine and injection supply costs per fully vaccinated child (2021 USD)* ^a^		
Bacille Calmette Guerin (BCG)	0.42	UNICEF (2020)
Hepatitis B	0.43	UNICEF (2020)
Oral Polio Virus	0.44	UNICEF (2020)
Pentavalent (DTP-HepB-Hib)	3.32	UNICEF (2020)
Inactivated polio virus	12.01	UNICEF (2020)
Pneumococcal conjugate	2.98	UNICEF (2020)
Rotavirus	5.47	UNICEF (2020)
Measles-Rubella	1.07	UNICEF (2020)
Diphtheria-tetanus-pertussis booster	0.33	UNICEF (2020)

Notes: IDSI = International Decision Support Initiative.

a Costs are adjusted from 2020 USD (as reported in UNICEF 2020) to 2021 USD.

#### Measurement of the impact of IMI on years of life saved due to deaths averted among children under the age of 5

To estimate the years of life saved (due to deaths averted among children under the age of 5), we calculated life expectancy at 2.5 years old in each of the five states in the study using vital statistics data from the Census of India (Census of India, 2022). We then multiplied this life expectancy by the estimated number of child deaths averted through IMI in each state and summed across states.

#### Measurement of the impact of IMI on DALYs averted

To estimate the number of DALYs averted through IMI, we combined condition-specific estimates of deaths averted with published estimates of the Years Lived with Disability and the Years of Life Lost per death for children under 5 years of age in India in 2017 (Institute for Health Metrics and Evaluation, 2018). Additional details are given in Supplementary Table S3.

#### Measurement of impact of IMI on cost-of-illness averted

To estimate the cost-of-illness averted through IMI, we multiplied condition-specific estimates of deaths averted by published estimates of the ratio of treatment costs averted to deaths averted by vaccination programmes in low- and middle-income countries in 2011–2020 (Ozawa *et al*., 2017). Additional details are given in Supplementary Table S4.

### Cost-effectiveness estimation

We estimated incremental cost effectiveness ratios (ICERs) for each major health outcome: the incremental cost (1) per dose delivered; (2) per zero-dose child reached; (3) per under-5 death averted; (4) per year of life saved and (5) per DALY averted, as described in the health outcomes section. We estimated ICERs from an immunization programme perspective (not incorporating costs-of-illness averted) and a societal perspective (incorporating cost-of-illness averted). Negative ICERs (resulting from negative health effects and positive cost estimates) were reported as ‘dominated’, indicating that the intervention would never be preferred to the status quo in these scenarios. We reported heterogeneity in outcomes by district and state, and used linear regression to examine associations between district characteristics [calculated from the 2016 Demographic & Health Surveys (Government of India, Ministry of Family Health and Welfare, International Institute for Population Sciences, 2015–2016)] and the incremental cost per incremental dose delivered.

We compared the incremental cost per DALY averted to a cost-effectiveness threshold of one per-capita gross domestic product (GDP) per DALY averted, as well as to empirically derived thresholds (Ochalek *et al*., 2018).

### Uncertainty estimation

We calculated 95% uncertainty intervals around study outcomes using a bootstrapping approach (Tibshirani and Efron, 1994). First, we drew 2000 samples of 40 districts (with replacement) from the 40 districts in the cost data sample and estimated the total cost for each of these samples. For each of these samples, we generated 1000 estimates of the total incremental doses delivered, reflecting uncertainty in the estimated treatment effect parameters in the CITS models, and producing 2 million estimates of each study outcome. We reported uncertainty as equal-tailed 95% uncertainty intervals and also generated cost-effectiveness acceptability curves (Fenwick *et al*., 2001) to estimate the probability that the intervention would be optimal for a range of cost-effectiveness thresholds.

### Sensitivity analysis

In the main analysis, we estimated the incremental doses delivered through IMI by summing the incremental dose estimates for each vaccine, e.g. pentavalent first dose (Penta 1), oral polio virus first dose (OPV1) and hepatitis B birth dose (HepB0). This approach allowed us to generate health impact estimates using the LiST model, since health impact varies across vaccines. As a sensitivity analysis to account for correlated effects across different vaccines, we fit a CITS model to the total number of vaccine doses delivered (the sum of all vaccines included in the study) and re-estimated the incremental cost per dose delivered using that model.

## Results

### Costs of IMI

The estimated incremental cost of IMI implementation in the 40 sampled districts was $13 708 000 (95% uncertainty interval: $10 560 000 to $17 351 000), including the costs of vaccines and injection supplies. Results excluding the costs of vaccines and injection supplies are shown in Supplementary Table S5.

### Health impact of IMI

**Figure 1.**
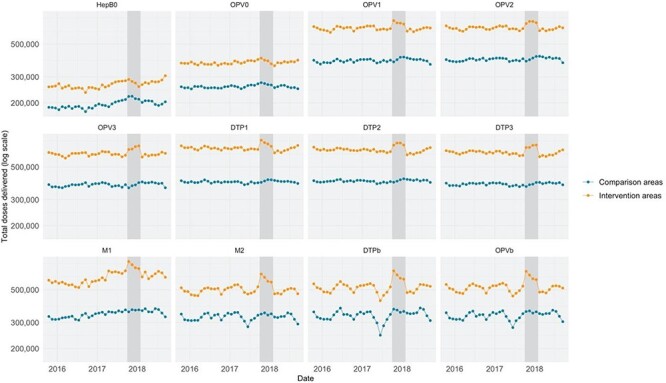
Trends over time in vaccine delivery in the five study states

Notes: Figure shows trends over time in the number of vaccine doses delivered in the five states in India from which the study sample was drawn. The dark grey area shows the period of IMI implementation. Blue points and lines indicate districts that did not participate in Intensified Mission Indradhanush (‘Comparison Areas’). Orange points and lines indicate districts that did participate in Intensified Mission Indradhanush (‘Intervention Areas’). Trends in doses delivered of the following vaccines are shown: Hepatitis B0 (HepB0), Oral Polio Vaccine (OPV) doses 1–3, Diphtheria-Tetanus-Pertussis (DTP) (or pentavalent vaccine) doses 1–3, Measles dose 1, Measles dose 2, Diphtheria-Tetanus-Pertussis booster (DTPb) and Oral Polio Vaccine booster (OPVb). Trends are adjusted for seasonality.

Figure 1 shows time trends in vaccine delivery in the five states from which the study sample was drawn, comparing districts that participated in IMI with districts that did not participate. The dark grey area shows the period of IMI implementation: for most vaccines, delivery is shown to increase during the implementation period and then return to earlier levels after the implementation period. The estimated number of incremental doses of the study vaccines delivered in the 40 sampled districts was 2 204 000 (−546 000 to 4 881 000). The estimated impact varied across vaccines. The smallest impacts were estimated for vaccines administered at birth apart from the BCG vaccine (49 000 incremental doses of OPV0 and 51 000 incremental doses of HepB0) and for booster doses (31 000 incremental doses of the OPV booster and 77 000 incremental doses of the DTP booster). The largest impacts were estimated for vaccines administered at 6 weeks of age (165 000 incremental doses of DTP1 and 139 000 incremental doses of OPV1). The estimated number of zero-dose children reached was 165 000 (−22 000 to 340 000).

We estimated that, by increasing immunization coverage in the sampled districts, IMI averted 1413 child deaths (−350 to 3129). Without discounting, this translated into 96 000 life years saved (−24 000 to 210 000) and 122 000 DALYs averted (−30 000 to 269 000) in the sampled districts. With discounting, the estimated number of life years saved was 38 000 (−9000 to 84 000) and DALYs averted was 42 000 (−10 000 to 93 000) in the sampled districts.

### Cost-effectiveness of IMI from an immunization programme perspective

**Figure 2.**
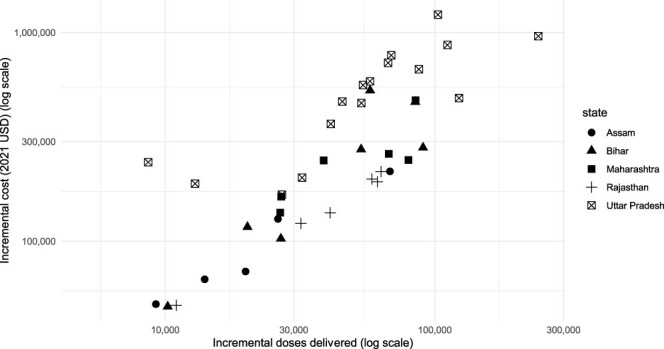
District-level estimates of incremental costs and incremental doses delivered

Notes: Figure shows the estimated incremental costs of Intensified Mission Indradhanush in each district (y-axis, on a log scale) and the incremental doses delivered in each district (x-axis, on a log scale) within the study sample. Costs are estimated from the immunization programme perspective. Vaccines and injection supplies are included. Different shapes represent different states.

We estimated that the incremental cost per dose delivered in the sampled districts (including vaccines) was $6.21 ($2.80 to dominated). There was substantial variation across districts in the estimated cost-effectiveness of IMI (Figure 2). District-level estimates for the incremental cost per dose ranged from $3.07 in Udaipur, Rajasthan, to $27.65 in Hapur, Uttar Pradesh. Districts with higher routine vaccine coverage in 2016 tended to have higher ICERs (Supplementary Table S2). There were no statistically significant differences in ICERs by urbanization levels, female literacy or wealth index.

Results also varied by state, from $3.43 per incremental dose delivered in the sampled districts in Rajasthan to $7.87 per incremental dose delivered in the sampled districts in Uttar Pradesh.

The incremental cost per zero-dose child reached in the sampled districts was $82.99 ($39.85 to dominated). District-level estimates of the incremental cost per zero-dose child reached ranged from $21.82 in Patna, Bihar, to $193.43 in Jaunpur, Uttar Pradesh.

Based on the results of the LiST model, we estimated that the incremental cost per under-5 death averted was $9701.35 ($4372.01 to dominated) and the incremental cost per life-year saved (through averting under-5 mortality) was $360.72 ($162.56 to dominated) in the sampled districts. We estimated that the incremental cost per DALY averted was $327.63 ($147.65 to dominated) in the sampled districts.

### Savings from averting costs of illnesses

We estimated that the total cost-of-illness averted by IMI in the sampled districts was $295 000 (−$73 000 to $654 000).

### Cost-effectiveness from a societal perspective

Accounting for averted costs of illness, the ICERs decreased to $6.09 ($2.67 to dominated) per incremental vaccine dose delivered, $81.20 ($38.08 to dominated) per incremental zero-dose child reached, $9492.46 ($4163.11 to dominated) per death averted, $352.95 ($154.79 to dominated) per life-year saved through averting under-5 mortality and $320.57 ($140.59 to dominated) per DALY averted.

### Uncertainty analysis

**Figure 3.**
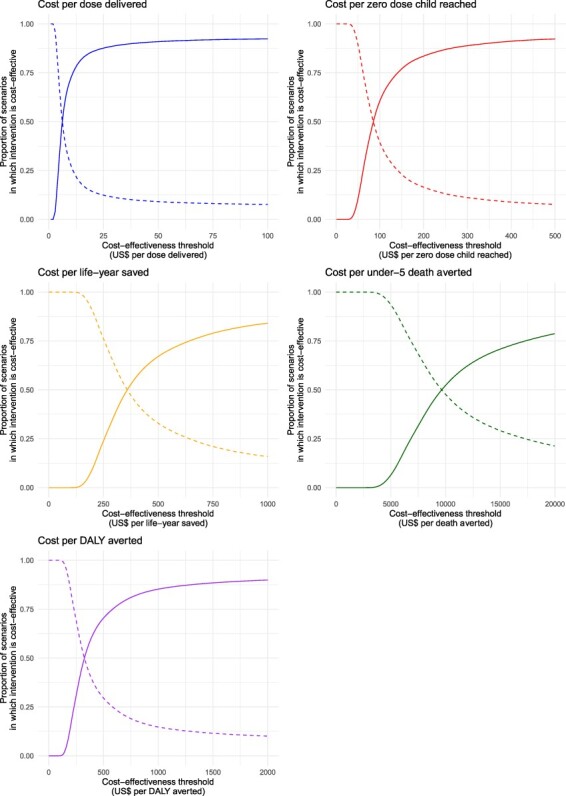
Cost-effectiveness acceptability curves

Notes: Figure shows the cost-effectiveness acceptability (CEA) curves for incremental cost per incremental dose delivered through Intensified Mission Indradhanush (IMI) the top-left panel (in blue), the incremental cost per zero-dose child reached through IMI in the top-right panel (in red), the incremental cost per incremental life-year saved in the middle left panel (in orange), the incremental cost per life saved in the botton right panel (in green) and the incremental cost per disability-adjusted life year (DALY) averted in the bottom left panel (in purple). The x-axis shows a theoretical cost-effectiveness threshold that a decision-maker may use. The y-axis shows the proportion of scenarios in which IMI would be considered cost-effective (solid line) or not cost-effective (dotted line) at this decision-making threshold. For example, if a decision-maker considers it cost effective to reach an incremental zero-dose child for $50, then IMI is most likely not cost-effective. If a decision-maker considers it cost-effective to save one life year for $963 (half of India’s annual gross domestic product, GDP, per capita in 2021), then IMI is cost-effective with a probability of 85%. If a decision-maker considers it cost-effective to avert one DALY for $1927 (India’s annual GDP per capita in 2021), then IMI is cost-effective with a probability of 89%.

Figure 3 shows cost-effectiveness acceptability curves for the four cost-effectiveness outcomes from an immunization programme perspective. Above a willingness-to-pay threshold of $359 per life-year saved, we estimated that there was a more than 50% probability that the intervention was cost-effective. At a willingness-to-pay threshold of 1× per-capita GDP per DALY averted, the intervention was estimated to be cost-effective with 90% probability. Using a threshold of $349 per DALY averted based on the estimated effect of changes in expenditure on morbidity and mortality (Ochalek *et al*., 2018), IMI was estimated to be cost-effective with 54% probability.

### Sensitivity analysis

When a single regression model was used to estimate the impact of IMI on total vaccine doses delivered (rather than separate models for each vaccine), the estimated total number of incremental vaccine doses delivered was 2 295 000 (−775 000 to 5 256 000) and the estimated ICER was $5.97 per incremental dose delivered ($2.60 to dominated), slightly lower than the main result.

### Patients and public involvement

Patients or the public were not involved in the design, conduct, reporting or dissemination plans of our research.

## Discussion

Following over a decade of minimal improvement in the coverage of traditional vaccines, the COVID-19 pandemic has significantly lowered routine immunization programme performance around the world, leaving more children unprotected and risking the resurgence of serious vaccine-preventable diseases such as measles, pertussis, polio, pneumococcal pneumonia and rotavirus diarrhoea. In this context, it is critically important to identify the best ways to increase immunization coverage with the limited available resources. This study examined the health and economic consequences of IMI, a PIRI intervention in India that delivered routine vaccines to an estimated 6 million children. In a sample of 40 districts across Assam, Bihar, Maharashtra, Rajasthan and Uttar Pradesh, our study suggests that IMI had a large impact on vaccine delivery and on the number of zero-dose children, had a large health impact—averting over 1400 child deaths in the study sample—and was cost-effective when using the threshold of one per-capita GDP per DALY averted. These findings are likely to be generalizable to the participating districts from which the sample was drawn, and illustrative of the cost-effectiveness of PIRI interventions in other similar settings.

Much of the published literature on the cost-effectiveness of vaccination considers the costs of vaccines alone or the costs of vaccine delivery at current levels of coverage. However, as countries work to increase access to vaccines, the focus must shift to the cost-effectiveness of interventions to improve coverage (Portnoy *et al*., 2021). While IMI was found to be cost-effective, the incremental cost per dose delivered through IMI was more than double the estimated cost of routine vaccine delivery in India reported in a recent cross-sectional study (Chatterjee *et al*., 2018). Furthermore, the incremental cost per dose delivered through IMI was higher in districts with higher baseline coverage. This is consistent with cost curves estimated in a previous systematic review (Ozawa *et al*., 2018). A possible explanation is that, as coverage gets higher, the remaining unvaccinated populations are harder to reach—e.g. due to remoteness, lack of awareness of vaccination or hesitancy towards vaccination—and require greater investment per child. This has implications for whether governments are adequately budgeting to address their zero-dose and under-vaccinated populations.

While there is little existing evidence on the cost-effectiveness of interventions to improve coverage (Munk *et al*., 2019), and these studies do not consistently report the same outcome measures, the cost-effectiveness of IMI can be compared with several related studies in India. The incremental costs of IMI per under-5 death averted and per DALY averted were higher than those found in an evaluation of an immunization programme strategy to reach unvaccinated children through awareness education of mothers in Uttar Pradesh (Powell-Jackson *et al*., 2018). The incremental cost of IMI per zero-dose child reached was also higher than that of a health information dissemination intervention in Uttar Pradesh that provided information about health services, including immunization services, to which the population was entitled (Pandey *et al*., 2007). Notably, both of these prior estimates come from trial settings, whereas we evaluated a programme implemented at scale. While IMI was primarily a supply-side intervention, it did include a social mobilization component; future work could focus on how to optimally design social mobilization efforts conducted as part of PIRI interventions.

Another approach for improving the cost-effectiveness of IMI could be to ensure that programme resources are effectively targeted towards reaching children who would not otherwise be reached by routine immunization services. When we compare our estimate of ‘incremental’ doses delivered to data on the number of doses delivered during IMI sessions, we find that approximately 63% of children reached were ‘incremental’, meaning that they would not have been vaccinated if IMI had not been implemented. However, approximately 37% of children vaccinated during IMI sessions were not incremental and would have been vaccinated even if IMI had not been implemented.

The strengths of this study include the use of empirical cost data and quasi-experimental estimates of programme impact and the use of a mathematical model to convert estimates of incremental doses delivered to health impact estimates.

This study has several limitations. First, there was a high degree of uncertainty in the estimated health impact of IMI (Clarke-Deelder *et al*., 2021), resulting in wide confidence intervals around cost-effectiveness results. As reported previously, estimates of the impact of IMI on vaccine delivery were large in magnitude but statistically insignificant for many vaccines, due to the noisiness of the HMIS data used in this analysis. However, we still found IMI to be cost-effective with a 90% probability using a 1× GDP per capita threshold. Second, our impact evaluation relied upon assumptions about how vaccine delivery would have changed over time in the absence of the programme. However, a wide range of sensitivity analyses testing these models found that the results were robust to a variety of model specifications (Clarke-Deelder *et al*., 2021). Third, our health impact and cost savings estimates rely on the assumptions of the LiST model and the published literature from which we extracted relevant parameters (Walker *et al*., 2013; Ozawa *et al*., 2017; Institute for Health Metrics and Evaluation, 2018). While the LiST model has been widely validated for child health impact estimates (Walker *et al*., 2013), some parameter values in our sample of under-performing districts may differ systematically from average values at the state or national level. In particular, life expectancy at 1 year and 5 years of age in our sampled districts may be lower than the average values in the states from which they were sampled. This could result in an upward bias in our estimates of health impact; however, such an approach is in line with the approach of using a ‘reference life expectancy’ in health impact analyses to avoid valuing lives in poorly performing places less than we value lives in higher-performing places. Fourth, the estimates of lives saved by vaccination were based only on mortality reductions in children, omitting the survival benefits of childhood immunization that accrue later in life. For example, infant vaccination against hepatitis B is protective against mortality from liver cancer later in life. As a result, our study is likely to underestimate the overall mortality impact of IMI. Fifth, our cost-effectiveness estimates from a societal perspective do not reflect patient-side cost savings related to reduced travel costs for immunization services during IMI; therefore, our results likely provide an upper bound on the true societal ICER. Sixth, our cost-effectiveness analysis used UNICEF-reported prices, but these prices are not India-specific. Finally, due to the nature of the HMIS, which does not disaggregate by population subgroup, we were not able to report the distributional impacts of IMI; however, unvaccinated children represent a priority group overall.

## Conclusions

Using a quasi-experimental analysis combined with an empirical costing study, we found that the large-scale implementation of PIRI was cost-effective in a sample of 40 districts in five states in India. While cost-effectiveness will vary with implementation approaches, scale and other contextual factors, PIRI interventions could be a cost-effective way to increase immunization coverage, reach zero-dose children and improve child health outcomes. There is a need for more research on the cost-effectiveness of approaches for improving coverage. Going forward, researchers should embed cost-effectiveness analyses in randomized trials, quasi-experimental studies and other implementation research of interventions to improve immunization coverage.

## Supplementary Material

czae024_Supp

## Data Availability

All study data are available on request from the authors.

## Reflexivity statement

This manuscript is the result of a collaboration among researchers affiliated with the Harvard T. H. Chan School of Public Health in Boston, Massachusetts, USA, the George Institute of Global Health in Delhi, India and the Bill & Melinda Gates Foundation in Seattle, Washington, USA and Delhi, India (at the time of project conception). Primary data collection in India was led by SC (based in Delhi during the time of this project) and analysis was led by EC (based in Boston during the time of this project). The author team is diverse in terms of gender identity and seniority. Team members based in India were involved in this study at all stages from conception through manuscript revision. However, the study team did not include individuals affected by the IMI programme or involved in its implementation, who might have been able to shed further light on reasons for its successes and failures.
