# Supplementary material for: Health impact and cost-effectiveness of expanding routine immunization coverage in India through Intensified Mission Indradhanush
Source: Health Policy Plan. 2024 Apr 3;39(6):583–92. doi: 10.1093/heapol/czae024 (PMC11145919; doi:10.1093/heapol/czae024)
Supplement: czae024_Supp [file czae024_supp.zip › suppl_data/IMI_CEA_Supplementary_Appendix_clean_March 27 2024.docx]

**Supplementary Appendix to**

**“Health impact and cost-effectiveness of expanding routine immunization coverage in India through Intensified Mission Indradhanush”**

**Table S1: Sources of estimates for incremental doses delivered**

| **Time point in Indian immunization schedule** | **Vaccine** | **Source for impact estimate** |
| --- | --- | --- |
| Birth | BCG | Estimated using controlling interrupted time-series |
| Birth | OPV0 | Estimated using controlling interrupted time-series |
| Birth | HepB0 | Estimated using controlling interrupted time-series |
| 6 weeks | Penta 1  (containing DTP1) | Estimated using controlling interrupted time-series |
| 6 weeks | OPV1 | Estimated using controlling interrupted time-series |
| 6 weeks | IPV1 | Assumed to be the same as Penta 1 (due to limited data availability) |
| 6 weeks | Rota1 | Assumed to be the same as Penta 1 (due to limited data availability) |
| 6 weeks | PCV1 | Assumed to be the same as Penta 1 (due to limited data availability) |
| 10 weeks | Penta 2  (containing DTP2) | Estimated using controlling interrupted time-series |
| 10 weeks | OPV2 | Estimated using controlling interrupted time-series |
| 10 weeks | Rota2 | Assumed to be the same as Penta 2 (due to limited data availability) |
| 10 weeks | PCV2 | Assumed to be the same as Penta 2 (due to limited data availability) |
| 14 weeks | Penta 3  (containing DTP3) | Estimated using controlling interrupted time-series |
| 14 weeks | OPV3 | Estimated using controlling interrupted time-series |
| 14 weeks | IPV2 | Assumed to be the same as Penta 3 (due to limited data availability) |
| 14 weeks | Rota3 | Assumed to be the same as Penta 3 (due to limited data availability) |
| 14 weeks | PCV3 | Assumed to be the same as Penta 3 (due to limited data availability) |
| 9 months | JE1 | Assumed to be the same as M1 (due to limited data availability) |
| 9 months | M1 | Estimated using controlling interrupted time-series |
| 16 months | DTP-b | Estimated using controlling interrupted time-series |
| 16 months | M2 | Estimated using controlling interrupted time-series |
| 16 months | OPV-b | Estimated using controlling interrupted time-series |
| 16 months | JE2 | Not included |
| 5-6 years | DTP-b2 | Not included |
| 10 years | TT | Not included |
| As soon as pregnancy is confirmed | TT1 | Not included |
| During pregnancy, four weeks after TT1 | TT2 | Not included |
| During pregnancy, if received 2 TT doses in a pregnancy within the past three years | TT-b | Not included |

Note: BCG = Bacillus Calmette–-Guérin; DTP = diphtheria-tetanus-pertussis vaccine; HepB = Hepatitis B; IPV = inactivated polio vaccine; JE = Japanese encephalitis vaccine; M = measles vaccine; OPV = oral polio vaccine; Penta = pentavalent vaccine, i.e., diphtheria-tetanus-pertussis-hepatitis B-*Haemophilus influenzae* type B; PCV = pneumococcal conjugative vaccine; Rota = rotavirus vaccine; TT = tetanus toxoid; 0 = birth dose; 1 = first dose; 2 = second dose; 3 = third dose; b = booster dose.

**Table S2: Regression analysis of district characteristics and incremental costs per dose delivered**

|  | **Coefficient**  **(95% Confidence Interval)** |
| --- | --- |
| Constant | -1.56  (-17.60 to 14.48) |
| State = Bihar | -2.06  (-9.59 to 5.48) |
| State = Maharashtra | -1.14  (-5.96 to 3.68) |
| State = Rajasthan | -3.02  (-10.23 to 4.17) |
| State = Uttar Pradesh | 4.79  (-0.20 to 9.76 ) |
| Urbanization level | 1.23  (-11.59 to 14.06) |
| DTP3 coverage | 13.04*  (1.36 to 24.72) |
| Female literacy rate | -2.24  (-24.64 to 21.14) |
| Wealth index | 0  (0.00 to 0.00) |

Notes: Table shows results from an ordinary least squares (OLS) linear regression model of the incremental cost per incremental dose delivered in each of the 40 sampled districts on district-level characteristics. The reference state is Assam. Urbanization level is measured from 0 to 1 as the portion of the district population living in an urban area. District characteristics are estimated using the 2016 Demographic and Health Survey (DHS) in India. DTP3 coverage is measured from 0 to 1 as the portion of children aged 12–23 months who had received a third dose of the diphtheria-tetanus-pertussis-containing vaccine prior to the survey. Female literacy rate is the portion of the adult female population who can read. Wealth index is the DHS-calculated wealth index based on household assets. *indicates statistical significance at the alpha = 0.1 level. **indicates statistical significance at the alpha = 0.05 level.

**Table S3: Conversion from deaths averted to DALYs averted**

| *1* | *2* | *3* | *4* | *5* | *6* | *7* |
| --- | --- | --- | --- | --- | --- | --- |
| **Vaccine-preventable disease** | **Under-5 deaths in India**  **(GBD 2017)** | **Under-5 YLLs in India (GBD 2017)** | **Under-5 YLDs in India (GBD 2017)** | **Ratio of YLDs to deaths** | **NPV of implied life expectancy** | **DALYs averted by IMI (Net Present Value in 2017)** |
| Diarrhea | 68,923 | 6,048,920 | 169,342 | 2.6 | 30.8 | 2,868 |
| Lower respiratory infections | 146,165 | 12,899,932 | 15,417 | 0.1 | 30.8 | 6,614 |
| Meningitis | 11,835 | 1,037,321 | 13,676 | 1.2 | 30.8 | 1,439 |
| Measles | 13,238 | 1,148,538 | 16,475 | 1.2 | 30.8 | 30,919 |

Notes: Table shows calculations of Disability Adjusted Life-Years (DALYs) averted through Intensified Mission Indradhanush (IMI). We extracted estimates of the total under-five deaths, Years of Life Lost (YLLs), and Years Lived with Disability (YLDs) in India in 2017 from the Global Burden of Disease (GBD) study (Columns 2, 3, and 4). To estimate the total number of DALYs averted, we first estimated the total number of YLDs averted. We did this by calculated the ratio of YLDs to deaths from vaccine-preventable diseases in India in 2017 (Column 5). We multiplied this ratio by the number of estimated deaths averted by IMI, estimated using the Lives Saved Tool. We assumed that YLDs were distributed over time in the same way as deaths (with all deaths and YLDs averted occurring over the period from 2018 through 2022). Next, we estimated the number of YLLs by multiplying the number of deaths averted by the net present value of the implied life expectancy from GBD (calculated by dividing the number of YLDs by the number of deaths, and applying a 3% discounting rate) (Column 6). We added together the YLLs and YLDs for each year from 2018 through 2022, and then discounted these to their Net Present Value in 2017 (Column 7). In this analysis, we used GBD estimates for diarrheal disease to represent rotavirus cases averted. We used GBD estimates for lower respiratory infections to represent pneumococcal pneumonia and pertussis cases averted.

**Table S4: Conversion from deaths averted to cost-of-illness saved**

| *1* | *2* | *3* | *4* | *5* |
| --- | --- | --- | --- | --- |
| **Vaccine-preventable disease** | **Deaths averted in LMICs 2011-2020 (Ozawa et al 2017)** | **Treatment costs averted in LMICs 2011-2020 (Ozawa et al 2017)**  **(USD 2021)** | **Ratio of treatment costs averted to deaths averted** | **Treatment costs averted through IMI (USD 2021)** |
| Rotavirus | 390,000 | $55,428,000 | 142.12 | $12,254 |
| Measles | 2,900,000 | $276,644,000 | 95.39 | $92,044 |
| Meningitis | 440,000 | $47,616,000 | 108.22 | $4,876 |
| Respiratory infections | 2,200,000 | $1,912,080,000 | 869.13 | $185,997 |

**Notes:** Table shows calculations of cost-of-illness averted through Intensified Mission Indradhanush (IMI). We extracted estimates of deaths averted and treatment costs averted in low- and middle-income countries (LMICs) due to vaccine-preventable conditions from Ozawa et al. (2017) and adjusted for inflation to USD 2021 (Columns 2 and 3). We then calculated the ratio of these estimates (Column 4). Next, we multiplied this ratio by the number of deaths averted due to IMI in each year in which the program had an estimated impact (2018 through 2022) to estimate the treatment costs averted, using a discount rate of 3%. We presented the treatment costs averted through IMI in USD 2021 (Column 5). For respiratory infections (specifically pertussis and pneumonia), we used Ozawa et al (2017) estimates for *Haemophilus influenzae* type B (Hib) because estimates were not reported for other respiratory infections.

**Table S5: Full cost-effectiveness results (USD 2021)**

| **Outcome** | **Immunization program perspective, excluding vaccine costs**  **(95% UI)** | **Immunization program perspective, including vaccine costs**  **(95% UI)** | **Societal perspective, excluding vaccine costs**  **(95% UI)** | **Societal perspective, including vaccine costs**  **(95% UI)** |
| --- | --- | --- | --- | --- |
| Incremental cost per dose delivered | $4.78  ($2.09 to dominated) | $6.21  ($2.80 to dominated) | $4.59  ($1.96 to dominated) | $6.09  ($2.67 to dominated) |
| Incremental cost per zero-dose child reached | $63.10  ($29.73 to dominated) | $82.99  ($39.85 to dominated) | $61.31  ($27.95 to dominated) | $81.20  ($38.08 to dominated) |
| Incremental cost per DALY averted | $249.09  ($110.27 to dominated) | $327.63  ($147.65 to dominated) | $242.03  ($103.22 to dominated) | $320.57  ($140.59 to dominated) |
| Incremental cost per life year saved | $274.24  ($121.41 to dominated) | $360.72  ($162.56 to dominated) | $266.48  ($113.65 to dominated) | $352.95  ($154.79 to dominated) |
| Incremental cost per life saved | $7,375.69  ($3,265 to dominated) | $9,701.35  ($4,372.01 to dominated) | $7,166.80  ($3,056.41 to dominated) | $9,492.46  ($4,163.11 to dominated) |
